# Malformations anorectales: revue de 6 ans aux Cliniques Universitaires de Lubumbashi

**DOI:** 10.11604/pamj.2021.38.64.22768

**Published:** 2021-01-20

**Authors:** Trésor Kibangula Kasanga, Didier Tshibangu Mujinga, Florent Tshibwid Zeng, Manix Ilunga Banza, Augustin Kibonge Mukakala, Eric Mbuya Musapudi, François Katshitsthi Mwamba, Prince Muteba Katambwa, Dimitri Kanyanda Nafatalewa, Christelle Ngoie Ngoie, Vincent De Paul Kaoma Cabala, Nathalie Dinganga Kapessa, Sébastien Mbuyi-Musanzayi

**Affiliations:** 1 Département de Chirurgie, Faculté de Médecine, Cliniques Universitaires de Lubumbashi, Université de Lubumbashi, Lubumbashi, République Démocratique du Congo,; 2 Service de Chirurgie, Hôpital Provincial de Référence Jason Sendwe de Lubumbashi, Lubumbashi, République Démocratique du Congo,; 3 Département de Chirurgie, Faculté de Médecine et Pharmacie, Cliniques Universitaires de Bukavu, Bukavu, République Démocratique du Congo

**Keywords:** Malformations anorectales, traitement, résultats, Anorectal malformation, treatment, results

## Abstract

Les malformations anorectales (MAR) sont des dysgénésies de la filière anogénitale comprenant plusieurs variétés anatomopathologiques. Leur diagnostic précoce permet une prise en charge précoce, laquelle réduit la morbi-mortalité y associée, surtout dans les pays en développement. L´objectif de cette étude est d´analyser les aspects épidémio-cliniques, thérapeutiques et évolutifs des malformations anorectales (MAR) aux Cliniques Universitaires de Lubumbashi. Il s´agit d´une étude descriptive transversale, ayant concerné 24 patients de 0 à 1 an admis dans notre service pour MAR. Sont exclus para cliniques dans notre série, les patients âgés de plus d'une année et non porteurs des examens demandés. Les données ont été recueillies sur base d´une fiche d´enquête reprenant les différents paramètres: l´âge au moment de la consultation, le sexe, le poids de naissance, la circonstance de découverte, la variété anatomique, le type de traitement et évolution des malades. La fréquence était de 24 cas (20,68%) des MAR sur 116 cas des malformations congénitales. L´âge médian était de 2 jours, le sexe-ratio de 1/3 en faveur des filles. L´occlusion intestinale était le diagnostic à l´admission le plus fréquent (50%). Les MAR basses étaient les plus fréquentes (11 patients soit 45,7%) dont 10 sans fistule. L´atrésie intestinale a été la malformation associée la plus fréquente (3 patients). L´anoplastie par abaissement abdomino-périnéal couplée aux dilatations anales postopératoires a été faite chez 13 patients, soit dans 54,1% des cas. Six patients sont décédés de causes inconnues et 6 autres ont été perdus de vues. Pour le résultat fonctionnel, sur les 12 des 24 patients pris en charge qui se sont présentés à la réévaluation 3 mois après sortie de l´hôpital, 3 d´entre eux ont présenté des signes d´incontinence, et 9 d´entre eux étaient continents parmi lesquels 8 étaient diagnostiqués avec MAR basse et 1 avec MAR haute. Les MAR sont une réalité dans notre milieu, cependant, le diagnostic n´est majoritairement posé qu´au décours des occlusions intestinales. Le taux des décès reste élevé et des mesures devrait être prises pour permettre des évaluations à long terme, lesquelles sont encore difficiles à faire vu le nombre des perdus de vue.

## Abstract (en)

Anorectal malformations (ARM) are developmental anomalies of the genitor anal elements, they represent a wide range of anomalies. An early diagnosis allows a better management as it reduces mortality associated with ARM, especially in developing countries. A prospective cross-sectional study has been carried, including patients from 0 to one year, admitted in our service for ARM. Twenty-four (24) patients have been considered. The median age was 2 days, the sex ratio 1/3 for female. Intestinal occlusions were the most frequent circumstances of diagnosis (50%), low ARMs were the most frequent, diagnosed in 11 patients (45.7%) of which 10 did not have any fistula. The most common associated malformation was intestinal atresia (3 patients). Anoplasty was done using abdominoperineal pull-through associated to anal dilatations in 13 patients (54.1%). Six patients died from unknown etiology and 6 were lost from the follow-up. Concerning the functional outcome, 12 of the 24 patients concerned by the study attended review and 3 of them presented signs of fecal incontinence, 9 of them were continents among whom 8 were diagnosed low ARM and one with high ARM. ARMs are encountered in our environment, nonetheless, the diagnosis is still made most of the time, during an intestinal occlusion. The mortality rate is still high and measures should be taken to allow long-term reviews, which will surely reduce the number of lost patients.

## Introduction

Les malformations anorectales constituent un groupe de malformations congénitales liées à un trouble de développement de l´intestin postérieur entrainant des anomalies touchant le rectum, le sphincter anal et le système urogénital et résultant en un large spectre de malformations allant des plus simples comme la sténose anale aux plus complexes, comme la persistance du cloaque [1]. La fréquence des MAR varie grandement selon les pays. Des études ayant compilé les données de plusieurs pays et rapportent une fréquence de 1/1.500 à 1/10.000 naissances vivantes [2]. Les pays en développement connaissent une fréquence élevée par rapport aux pays développés et cela serait attribuable, entre autres, au meilleur fonctionnement des systèmes de santé, permettant de réduire les diagnostics manqués [3]. La majorité des MAR a une étiologie probablement multifactorielle, il y a une interaction entre les facteurs génétiques et environnementaux. Tout trouble des gènes régulant le développement de l´intestin postérieur (Wnt, Hox, Shh, Gli2, Bmp 4, Fgf et CDX1) est susceptible de contribuer à la genèse des MAR [4]. Parmi les facteurs environnementaux reconnus, on peut citer, avant la grossesse, les médicaments anti-asthmatiques, les hypnotiques et les benzodiazépines, le tabagisme parental, les techniques de reproduction assistée, les pneumopathies chroniques, l´obésité et le diabète [5]. Pendant la grossesse, l´absence de nausée et de vomissement, la fièvre au premier trimestre, les déficits en vitamine A et en acide folique, la répétition des infections urinaires et les maladies thyroïdiennes [6]. Les deux sexes semblent être atteints de la même manière, même si certaines études trouvent une légère prédominance en faveur d´un des deux et ce, selon les pays. Cependant, certains types de MAR sont plus fréquents dans l´un des sexes, comme les MAR avec fistule périnéale chez les filles [7].

Les MAR peuvent être classées en formes syndromiques et isolées. Aujourd´hui encore, les chirurgiens utilisent des terminologies différentes en ce qui concerne la classification des MAR isolées. Cela est lié à la variété des présentations cliniques et aux nombreuses tentatives de classification, depuis 1835 avec la première tentative par Amussat. Ensuite, plusieurs ont vu le jour, dont les plus populaires sont: le système de classification de Ladd et Gross (1934), la classification internationale de Melbourne catégorisant (1970), la classification de Wingspread (1984), la classification de Stephens (1986), la classification de Peña (1990). En 2005, les chirurgiens pédiatres réunis à Krickenbeck trouvent un consensus pour utiliser la classification proposée par Holschneider, désormais appelée classification de Krickenbeck [1,8]. L´examen néonatal permet, en général, de reconnaître différentes variétés des MAR. Parfois, le diagnostic n´est posé que devant des signes de complications, à distance de la période néonatale. Dès que le diagnostic est retenu, des examens paracliniques sont réalisés d´abord pour classer le type de MAR et ensuite pour rechercher des malformations associées [9]. Dans presque la moitié des cas, les MAR s´accompagnent de malformations associées, dont les plus fréquentes sont cardiovasculaires (communication interauriculaire, persistance du canal artériel et communication interventriculaire), urogénitales (hydronéphrose et hypospadias), digestives (atrésie de l´œsophage et mégacôlon) et tant d´autres (hernie inguinale, polydactylie, déformation de la colonne vertébrale, fentes labiopalatines) [10].

Le but du traitement chirurgical des MAR est la reconstitution d´une anatomie la plus naturelle possible pour permettre une continence fécale et urinaire ainsi qu´une fonction sexuelle satisfaisante en lésant le moins de structures possibles. En général, 3 opérations sont nécessaires, mais cela varie selon le sexe et le type de MAR [11]. La première intervention est une colostomie protective, qui est ensuite suivie d´une anoplastie. Pour cette dernière, l´abaissement abdominopérinéale se fait plusieurs techniques dont la plus populaire est l´anorectoplastie postérosagittale, introduite depuis 1982 par Alberto Peña. Le double abord périnéo-abdominal assisté par la laparoscopie a montré beaucoup d´avantages pour certains types de MAR [12]. La dernière intervention est la fermeture de la colostomie. Pour prévenir les structures, des dilatations anales sont recommandées dès la deuxième semaine post opératoire [13]. La mortalité reste fortement liée aux malformations associées, mais aussi et surtout en ce qui concerne les pays en développement, au retard de diagnostic et de prise en charge [14]. Les troubles du transit intestinal restent les plus fréquentes complications suivant la chirurgie. La constipation est plus fréquente dans les MAR basses alors que l´incontinence est plus fréquente dans les MAR hautes. Le suivi à long terme doit inclure les éléments urologiques et gynécologiques [11]. Eu égard à ce qui précède, nous avons entrepris cette étude dans notre milieu pour avoir des données en rapport cette pathologie sur plan épidémio-clinique, anatomopathologique, thérapeutique et évolutif.

## Méthodes

Il s´agit d´une étude descriptive transversale prospective menée aux Cliniques Universitaires de Lubumbashi pendant la période allant du 01^er^ janvier 2013 au 31 décembre 2018. L´étude a concerné tous les patients de 0 à 1 an, diagnostiqués avec une MAR et traités aux CUL. Au total, 24 enfants ont été retenus. Sont exclus dans notre série patients âgés de plus d´une année et non porteurs des examens para cliniques demandés Les données ont été recueillies sur base d´une fiche d´enquête reprenant les différents paramètres: l´âge au moment de la consultation, le sexe, le poids de naissance, la circonstance de découverte, la variété anatomique, le type de traitement et évolution des malades. Les éléments recueillis ont été encodés et analysés avec les logiciels Epi Info 7.0.9.7 et Excel de Microsoft Office 2016. Les variables statistiques utilisées sont: la moyenne, la médiane, l´écart-type et la proportion en pourcentage.

## Résultats

La fréquence était de 24 cas (20,68%) de MAR sur 116 cas des malformations congénitales. Sur l´ensemble des 24 patients pris en compte dans notre étude, la médiane d´âge était de 2 jours, les filles ont largement dominé l´échantillon avec 18 patientes contre 6 patients, soit un sexe-ratio H/F de 1/3. Le poids de naissance médian était de 2800 grammes, avec un minimum de 2500 grammes et un maximum de 3500 grammes. En ce qui concerne la circonstance de découverte de la maladie, l´occlusion intestinale était la situation la plus fréquente (50%), suivie d´émission des selles à travers le méat urétral (fistule urinaire) ou à travers un pertuis périnéal (fistule périnéale) anormalement localisé (29,2%). La découverte à la naissance a été faite dans 20,8% des cas Tableau 1. Selon la classification de Stephens, les MAR hautes ont été rencontrées chez 8 patients (33,3%) parmi lesquels 5 avaient des fistules. Aucune MAR intermédiaire n´a été diagnostiquée et les formes basses ont été les plus fréquentes, retenues chez 11 patients (45,7%) parmi lesquels 10 étaient sans fistule. Les types non spécifiques ont été enregistrés chez 5 patients, toutes sans fistule (Tableau 2).

**Tableau 1:** circonstances de diagnostic

Circonstances de decouverte	Effectif	Pourcentage
Diagnostic à la naissance	5	20,83
Occlusion intestinale	12	50,00
Fistule urinaire	2	8,33
Fistule périnéale	5	20,83
Total	24	100,00

L’occlusion intestinale représente la circonstance de découverte la plus fréquente à 50%.

**Tableau 2:** variété anatomopathologique

Variété de MAR	Présence de fistule				Total	
	Présence		Absence			
	Effectif	Pourcentage	Effectif	Pourcentage	Effectif	Pourcentage
Haute	5	62,5	3	37,5	8	33,3
Intermédiaire	0	0	0	0	0	0
Basse	1	9,1	10	90,9	11	46,30
Non spécifiée	0	0	5	100	5	21,00
Total	6	33,3	18	66,7	24	100

La variété haute est la plus associée aux fistules à 62,5%

Au cours de notre étude, deux malformations associées ont été identifiées: l´atrésie intestinale, chez 3 patients, soit 12,5% de notre échantillon et la hernie ombilicale, chez 1 seul patient, soit 4,1%. Les malformations cardiaques n´ont pas été explorées sur base d´un examen clinique cardiovasculaire normal. Le traitement chirurgical le plus pratiqué était l´anoplastie couplée aux dilatations anales postopératoires. Cette méthode a été faite chez 13 patients, soit dans 54,1% des cas. Les colostomies en première intention ont été réalisées chez 6 patients (25% des cas) et 5 autres patients (20,8% de l´échantillon), avaient bénéficié des dilatations anales seules (Tableau 3). Dans notre série, 12 patients, soit 50% de notre population d´étude, avaient bien évolué; 6 sont décédés de causes diverses (anesthésie chez des patients pris tardivement, les infections surtout pulmonaires et le sepsis), et 6 autres ont été perdus de vues. Pour le résultat fonctionnel, 50% de nos patients s´étaient présentés à la réévaluation et 3 d´entre eux ont présenté des signes d´incontinence (souillure fréquente, érythème fessier et sphincter atone au toucher rectal), alors que 9 d´entre eux étaient continents parmi lesquels, dont 8 avait été diagnostiqués avec MAR basse et 1 avec MAR haute (Tableau 4).

**Tableau 3:** traitement chirurgical

Traitement	Effectif	Pourcentage
Dilatations anales	5	20,8
Anoplastie + dilatations	13	54,2
Colostomie	6	25

Traitement chirurgical le plus réalisé, anoplastie + dilatations à 54,2%

**Tableau 4:** résultat fonctionnel

Variété de MAR	Incontinence	Continence
MAR haute	3	1
MAR basse	0	8
Total	3	9
Pourcentage	25	75

La variété haute est plus associée à l’incontinence anale à 25%

## Discussion

Notre série a trouvé une nette prédominance féminine, avec une sex-ratio de 1/3. Ceci est différent de ce qui est généralement rapporté dans plusieurs études, qui identifient presque toujours une prédominance masculine [15]. Cependant, certaines études ont rapporté une prédominance féminine [16]. Nous pensons que ces différences sont liées aux différences des tailles des échantillons. La plus fréquente circonstance de découverte a été la survenue d´une occlusion intestinale (soit 50% des cas). Ceci contraste avec le fait que les MAR devraient normalement être diagnostiquées facilement à la naissance, cela permettrait de réduire les complications qui sont plus fréquentes en cas de retard de diagnostic. Cependant, il est assez commun de manquer le diagnostic au premier examen et de ne reconnaître les MAR qu´après 24 heures, avec la présence des signes d´occlusion dont le ballonnement abdominal. Dans les pays en développement, la naissance en dehors du milieu hospitalier fait manquer un examen néonatal initial et retarde le diagnostic et par ricochet le traitement [17,18]. Dans notre série, les MAR basses étaient plus représentées avec 45,9%, suivies des MAR hautes et des formes spécifiques. Aucune malformation intermédiaire n´a été diagnostiquée. Notre tendance rejoint celle de Ngom *et al*. qui avait rapporté 44,3% pour les MAR basses, 29% pour les MAR hautes, 13,9% pour les MAR non spécifiques et 9,4% pour les MAR intermédiaires, les moins représentée de sa série aussi. Luhiriri *et al*. ont trouvé les MAR hautes en majorité (56,6%), suivies des MAR basses (33,2%) et des MAR intermédiaires (13,2%). Ces différences pourraient être liées aux différences de taille des échantillons [18].

Notre série a identifié des malformations associées chez 4 patients, soit 16,7% de notre population d´étude. Ce pourcentage est inférieur à ce qui est classiquement décrit, soit presque dans 50% des cas. Dans presque la moitié des cas, les MAR s´accompagnent de malformations associées, dont les plus fréquentes sont cardiovasculaires (CIA, CAP et CIV), urogénitales (hydronéphrose et hypospadias), digestives (atrésie de l´œsophage et mégacôlon) et tant d´autres (hernie inguinale, polydactylie, déformation de la CV, Fentes Labiopalatines) [10,19]. Cela pourrait s´expliquer par le fait que notre échantillon est moins important. Des recherches menées avec un échantillon moins important ont trouvé des fréquences de malformations associées moins importantes proche: 13,8% à 28% [18,20]. Selon la population d´étude, les malformations associées les plus fréquemment rencontrées varient. Dans notre étude, les malformations digestives ont été les plus rencontrées, avec en tête, les atrésies intestinales rencontrées dans 12,5% des cas. Dans la littérature, des atrésies intestinales ont déjà été rapportées avec une fréquence de 4 à 5% [21]. Plusieurs scores d´évaluation de la continence ont été proposés par différents auteurs [22,23]. Pour notre étude, la restriction aux moins d´un an a fait que nous nous sommes proposés d´utiliser certains indicateurs de la continence anale en postopératoire. Nous avons considéré la souillure continuelle, la présence d´un érythème fessier et l´atonie du sphincter anal comme étant des éléments en faveur d´une incontinence. Ainsi, notre série a trouvé que les patients étaient continents dans 75% des cas, ce qui est proche des 66,6% trouvés par Luhiriri *et al*. en se basant sur les mêmes critères, Ngom *et al*. ont trouvé 70% des patients avec des éléments favorables à l´incontinence. Cette différence pourrait s´expliquer par le fait que dans la population de Ngom *et al*. les MAR intermédiaires et hautes, lesquels sont sujets à une plus grande fréquence d´incontinence [18,24]. Le nombre de décès, dans notre série est de 6, soit 25% de l´échantillon. Cela est largement supérieur à la mortalité des centres des pays développés. Dans ces pays, la survie globale atteint 88% [16]. Cette différence pourrait être liée au fait que les patients en milieu Africain sont pris en charge tardivement, ce qui augmente les complications desquelles découlent une grande mortalité.

## Conclusion

Les MAR sont une réalité dans notre milieu. A la différence des pays développés où elles sont prises en charge à temps, le retard de prise en charge chez nous altère sérieusement le pronostic avec un taux élevé de mortalité. Le quart de notre population d´étude est perdue de vue, ce qui illustre davantage le défi pour le chirurgien à manager les MAR dans notre milieu, avec peu de chances d´effectuer un suivi à long terme.

### Etat des connaissances sur le sujet

- Dans les pays en voie de développement, les MAR sont diagnostiquées tardivement par voie des conséquences la morbi-mortalité y reste élevée;
- Les MAR diagnostiquées et prise en charge précocement, le pronostic reste bon;
- Les MAR hautes s´accompagnent souvent de l´incontinence anale.

### Contribution de notre étude à la connaissance

- Les circonstances de découvertes dans notre milieu sont dominées par la survenue de l´occlusion intestinale et non à l´accouchement comme partout ailleurs;
- La limite dans l´évaluation objective de l'appareil sphinctérien par manque de la manométrie anale dans notre milieu, nous nous sommes proposés d´utiliser certains indicateurs de la continence anale en postopératoire, la souillure continuelle de la région anale, la présence d´un érythème fessier et l´atonie du sphincter.
